# The relationship and influencing factors of critical thinking and medical ethical decision-making among pediatric medical students

**DOI:** 10.1097/MD.0000000000039865

**Published:** 2024-09-20

**Authors:** Hongxing Dang, Shaojun Li, Jing Li

**Affiliations:** a Department of Pediatric Intensive Care Unit, Children’s Hospital of Chongqing Medical University, Chongqing, China; b Ministry of Education Key Laboratory of Child Development and Disorders of China, Chongqing, China; c China National Clinical Research Center for Child Health and Disorders, China International Science and Technology Cooperation Base of Child Development and Critical Disorders, Chongqing, China; d Department of Emergency Medicine, Children’s Hospital of Chongqing Medical University, Chongqing, China.

**Keywords:** critical thinking dispositions, medical education, medical ethics, pediatrics, undergraduates

## Abstract

Pediatric diseases possess unique characteristics, requiring pediatricians to have strong critical thinking skills and sound ethical decision-making abilities. This study aims to investigate and analyze the critical thinking dispositions of pediatric medical students and their impact on ethical decision-making levels, and to propose suggestions for improving teaching methods. A cross-sectional study design was adopted, using the Chinese version of the California Critical Thinking Disposition Inventory (CCTDI-CV) and an ethical decision-making questionnaire. An online survey was conducted among 240 pediatric medical students at Chongqing Medical University, collecting participants’ basic demographic information. The study described the CCTDI-CV scores and ethical decision-making questionnaire scores (mean ± standard deviation), with distribution and trend analyses performed using *t* tests and *H*-tests. Pearson correlation analysis was used to examine the relationship between the 2, and regression analysis was conducted to explore factors influencing ethical decision-making abilities. A total of 229 students (95.4%) completed the survey. The overall average score of critical thinking disposition among pediatric medical students was 287.96 ± 39.09, with 139 students (60.70%) demonstrating positive or highly positive critical thinking dispositions. Ethical decision-making abilities were excellent in 85 students (37.12%). There was a significant positive correlation between critical thinking abilities and ethical decision-making abilities (*R* = 0.774, *P* < .001), particularly with analysis abilities, systematic abilities, and cognitive maturity showing higher correlations with total ethical decision-making scores. CCTDI-CV scores had a significant positive impact on ethical decision-making levels (*P* < .001), with factors such as family background and high school performance also significantly influencing ethical decision-making abilities (*P* < .001). Chinese pediatric medical students generally exhibit strong critical thinking and ethical decision-making abilities. Critical thinking plays a crucial role in medical ethical decision-making, with family background and high school performance being important influencing factors. Educators should focus more on developing multidimensional critical thinking skills to enhance students’ ethical decision-making abilities, thereby improving overall healthcare service quality. The study results also provide new perspectives for international pediatric medical educators.

## 1. Introduction

With the development of modern medicine, the importance of critical thinking and medical ethical decision-making in medical practice has become increasingly prominent. Medical students need not only to master solid medical knowledge but also to possess a high level of critical thinking skills and rigorous ethical judgment abilities. Critical thinking is a mode of thinking based on logical and objective analysis, which can help medical students make wise decisions in complex clinical environments.^[1]^ Medical ethical decision-making, on the other hand, is an essential issue that healthcare professionals must face in clinical practice. It requires them to consider medical ethical principles and practical circumstances when handling situations involving patient rights and moral norms, balancing respect for patient and family rights, maximizing benefits, and ensuring fairness.^[2]^ The core position of medical ethics in undergraduate medical education has been widely recognized, and systematic ethical education can help students make better decisions when confronted with complex clinical and legal issues.^[3]^

Ethical decision-making is not merely the application of knowledge but a concrete manifestation of critical thinking in practice. Critical thinking can enhance moral reasoning and guide physicians in fulfilling their ethical responsibilities, thereby improving the quality of healthcare.^[4]^ In pediatric medicine, ethical decision-making is particularly complex and sensitive.^[5]^ Given the limited expressive and judgmental abilities of pediatric patients, medical decisions often rely on the opinions of guardians and the professional judgment of physicians. Physicians must use robust critical thinking to assess the potential impacts of each decision, ensuring that their ethical decisions meet medical standards while fully respecting the rights of the patients and their families.^[6]^

In Western countries, medical education emphasizes fostering students’ independent thinking and clinical decision-making abilities.^[7]^ In contrast, traditional medical education in China tends to focus more on the transmission and reception of knowledge. Studies have shown that Chinese medical students often exhibit contradictory critical thinking dispositions, indicating significant diversity and potential for improvement.^[8]^ In comparison, healthcare professionals in other countries display more positive and confident critical thinking dispositions.^[9–11]^ These differences may be attributed to variations in educational systems, cultural backgrounds, and teaching methods and they may have profound implications for the ethical decision-making tendencies of medical students.

Understanding the impact of critical thinking on ethical decision-making abilities can help educators adjust their approaches. However, research on the relationship between critical thinking and medical ethical decision-making and its influencing factors remains limited, especially for pediatric medical students. There is also a lack of empirical studies specifically targeting pediatric medical education. Therefore, this study aims to analyze the role of critical thinking in the ethical decision-making process of pediatric medical students and its influencing factors. It also seeks to explore how education and training can enhance pediatric medical students’ critical thinking and ethical judgment levels, providing a theoretical basis for the further development of medical education.

The Department of Pediatrics at Chongqing Medical University is one of China’s major training bases for pediatricians, having trained over half of the country’s pediatricians (>4000) in the past 20 years. Chongqing Medical University has not only influenced pediatric medical education in China but also provided significant references and experiences for global pediatric medical education. Thus, this study will conduct a cross-sectional survey on the critical thinking dispositions and ethical decision-making abilities of pediatric medical students at Chongqing Medical University. Firstly, it will describe the demographic characteristics of the sample and their performance in critical thinking and ethical decision-making abilities. Secondly, it will explore the relationship between critical thinking and ethical decision-making levels through correlation analysis. Finally, it will use regression analysis to examine the effects of critical thinking abilities and demographic characteristics on ethical decision-making levels, revealing key factors influencing ethical decision-making levels. This study aims to provide valuable insights for improving pediatric medical ethics education, not only in China but globally.

Through this study, we hope to reveal the current state of critical thinking and medical ethical decision-making among pediatric medical students, identify key factors influencing ethical decision-making, provide valuable references for medical educators, and offer new perspectives and methods for training medical students. By emphasizing the cultivation of ethical decision-making abilities in the teaching process, we aim to better prepare future pediatricians to face the complexities of clinical practice, thereby enhancing the quality of healthcare services provided by future pediatricians.

## 2. Methods

### 2.1. Study subjects and procedure

This study combined the Critical Thinking Disposition Inventory and the Ethical Decision-Making Questionnaire, conducting an online survey through the “Questionnaire Star” platform (https://www.wjx.cn/) from September to December 2023. A cross-sectional survey was conducted among 240 pediatric students at Chongqing Medical University. Inclusion criteria were: (1) students enrolled in the undergraduate pediatric program; (2) willingness to participate in the survey with no history of participating in similar assessments. Exclusion criteria included incomplete questionnaire responses.

After interacting with students in the classroom, researchers explained the study’s purpose, survey characteristics, and testing methods: normal completion time was 20 to 30 minutes, and tests completed in <15 minutes were considered invalid; each participant was allowed to submit only once, with no repeated submissions; participants were instructed not to discuss the test content with others. All participants remained anonymous, and survey results were used solely for educational research to improve teaching methods. Students were free to choose whether or not to participate in the survey, and participants could withdraw from the survey at any time at their discretion. The study was reviewed and approved by the Institutional Review Board of Chongqing Medical University. Since the survey was anonymous, voluntary, and did not adversely affect participants’ rights and health, the requirement for written informed consent was waived.

### 2.2. California Critical Thinking Disposition Inventory and ethical decision-making questionnaire testing and data collection

The survey utilized the Chinese version of the California Critical Thinking Disposition Inventory (CCTDI-CV).^[12]^ This self-report questionnaire is derived from the CCTDI^[13]^ and uses a 6-point Likert scale, encompassing 7 subdimensions: truth-seeking, open-mindedness, analysis ability, systematicity, critical thinking self-confidence, inquisitiveness, and cognitive maturity. Each subdimension consists of 10 items, with scores ranging from “1” (strongly disagree) to “6” (strongly agree). The total score range of the questionnaire is 70 to 420, with higher scores indicating a stronger disposition towards critical thinking. Subdimension scores above 40 and a total score above 280 are assessed as positive dispositions. Critical thinking dispositions are categorized into 4 groups: relatively negative (≤210), ambivalent (211–279), positive (280–349), and highly positive (≥350).^[14]^ The overall content validity index of the CCTDI-CV is 0.85, matching that of the original English version, with a Cronbach alpha coefficient of 0.71.^[13]^

We also used the commonly applied ethical decision-making questionnaire from our institution, which includes 6 subdimensions: informed consent, resource allocation, patient privacy, patient autonomy, conflict of interest, and ethical decision-making in emergencies. Each subdimension is designed with a case scenario containing 2 sequential questions, with different answer choices scoring from 0 to 3 points. The total score of the questionnaire is 36 points, with higher scores indicating stronger ethical judgment and decision-making abilities, categorized as excellent (30–36), good (24–29), fair (18–23), and poor (≤17). This questionnaire was revised by experts from various fields, including medical ethics, psychology, and education, and demonstrated good content validity. We also tested the internal consistency and suitability of the scale’s items, resulting in a Cronbach alpha coefficient of 0.78 and a Kaiser-Meyer-Olkin value of 0.9, indicating good internal consistency and suitability for factor analysis.

Additionally, we collected basic demographic information from the participants, including gender, family background (urban or rural), only-child status, high school academic ranking, and parents’ highest education level.

### 2.3. Statistical analysis

Statistical analyses were performed using SPSS software version 23.0. Descriptive statistics, including frequencies, means, and standard deviations, were first conducted to understand the basic characteristics of the data. To assess the distribution and categorical comparison of critical thinking dispositions and ethical decision-making levels, trend analyses were performed using *t* tests and Kruskal–Wallis *H* tests. The relationships between the total CCTDI-CV scores, its subdimension scores, and ethical decision-making scores were analyzed using Pearson correlation analysis. The impacts of various critical thinking subdimensions and other factors on ethical decision-making levels were examined using multivariate regression analysis. All tests were considered statistically significant at a *P*-value of <.05.

## 3. Results

### 3.1. Characteristics of the study subjects

After distributing electronic questionnaires to all 240 students, a total of 229 pediatric students (95.4%) participated in the critical thinking survey and submitted complete questionnaires. Among them, 135 were female, accounting for approximately 58.95% of the total participants. Students from rural family backgrounds numbered 53 (23.14%), while those from county-level or higher urban family backgrounds numbered 176 (76.86%). Approximately 40.61% were only children, totaling 93 students. Regarding high school academic performance, 87 students (37.99%) ranked in the top 10% of their grade, 63 students (27.51%) ranked in the top 10% to 20%, and 58 students (25.33%) ranked in the top 20% to 30%. There were 89 students (38.86%) whose parents had attained a bachelor’s degree or higher, including 4 students whose parents held master’s or doctoral degrees.

### 3.2. Performance of pediatric students on the CCTDI-CV and ethical decision-making abilities

The overall average score of pediatric students on the CCTDI-CV was 287.96 ± 39.09. Regarding critical thinking dispositions, 4 students (1.75%) exhibited a negative disposition (≤210), 86 students (37.55%) were in an ambivalent state (211–279), 128 students (55.90%) demonstrated a positive disposition (280–350), and 11 students (4.80%) showed a highly positive critical thinking disposition (≥350). For ethical decision-making abilities, the average score was 27.72 ± 5.20. In terms of performance, 85 students (37.12%) were rated as excellent (30–36), 102 students (44.54%) were rated as good (24–29), 39 students (17.03%) were rated as fair (18–23), and only 3 students (0.01%) were rated as poor (≤17).

### 3.3. Distribution of ethical decision-making levels and critical thinking dispositions

The higher the ethical decision-making scores of pediatric students, the higher their CCTDI-CV scores (*P* < .001). Table 1 presents the distribution of CCTDI-CV scores across different ethical decision-making scores groups.

**Table 1:** Distribution of ethical decision-making scores and CCTDI-CV scores (n, %).

Ethical decision-making score	CCTDI-CV scores					Statistic	*P*
	≤210	211–279	280–349	≥350	Mean ± SD		
Excellent (30–36)	0 (0.0)	7 (8.24)	68 (80.0)	10 (11.76)	316.62 ± 18.75	*H* = 90.84	＜.001
Good (24–29)	0 (0.0)	48 (47.06)	53 (51.96)	1 (0.98)	281.44 ± 16.17		
Fair (18–23)	1 (2.56)	31 (79.49)	7 (17.95)	0 (0.0)	251.92 ± 13.41		
Poor (≤17)	3 (100.0)	0 (0.0)	0 (0.0)	0 (0.0)	166.00 ± 14.14		

CCTDI-CV = Chinese version of the California Critical Thinking Disposition Inventory.

### 3.4. Correlation between CCTDI-CV subdimension scores, total scores, and ethical decision-making total scores

**Figure 1.**
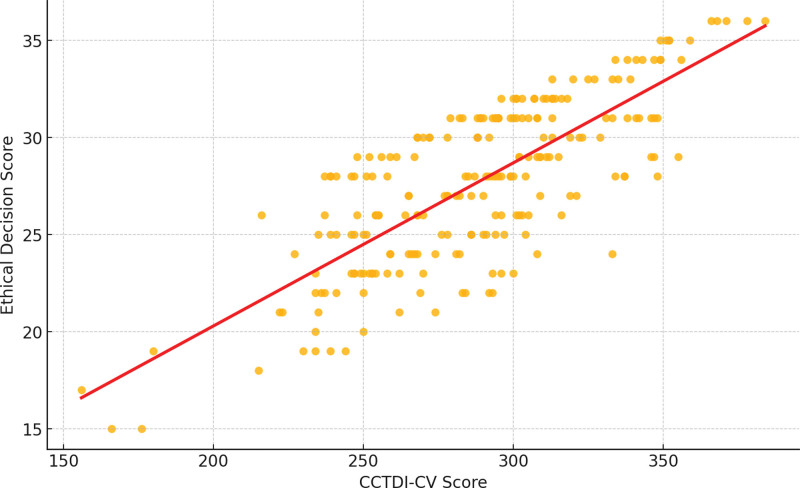
Correlation between total CCTDI-CV scores and ethical decision-making scores. Pearson correlation test, *R* = 0.774, *P* < .001. CCTDI-CV = Chinese version of the California Critical Thinking Disposition Inventory.

In the CCTDI-CV, the subdimension scores for open-mindedness, systematicity, inquisitiveness, and cognitive maturity all exceeded 40 points. Correlation analysis showed that all CCTDI-CV subdimensions were positively correlated with the total ethical decision-making scores (Table 2), with analysis ability, systematicity, and cognitive maturity having higher correlations (*R* > 0.6). The highest correlation was observed between the total CCTDI-CV score and ethical decision-making (*P* < .001) (Fig. 1).

**Table 2:** Correlation between CCTDI-CV subdimensions and ethical decision-making total scores.

CCTDI-CV subdimension	Scores	Correlation coefficient (*r*)
Truth-seeking	39.95 ± 8.68	0.552
Open-mindedness	43.62 ± 8.67	0.558
Analysis ability	38.86 ± 7.40	0.616
Systematicity	39.46 ± 4.87	0.629
Critical thinking self-confidence	42.91 ± 7.78	0.566
Inquisitiveness	40.06 ± 6.14	0.538
Cognitive maturity	43.09 ± 7.10	0.707

*Note*: Pearson correlation test indicates that all correlations are significant at the *P* < .01 level.

CCTDI-CV = Chinese version of the California Critical Thinking Disposition Inventory.

### 3.5. Relationship between demographic characteristics and ethical decision-making scores

The study found significant correlations between several demographic characteristics and ethical decision-making scores. Students from rural areas had lower average scores compared to those from non-rural areas. Only children had higher average scores than students with siblings. Students with higher high school academic performance and parents with higher education levels (bachelor’s degree or above) also scored higher (*P* < .05). Female students had higher average scores than male students, although this difference was not statistically significant. Table 3 presents the distribution of ethical decision-making scores among pediatric students across different demographic characteristics.

**Table 3:** Distribution and comparative analysis of ethical decision-making scores among pediatric students by demographic characteristics (n, %).

	Excellent (30–36)			Good (24–29)		Fair (18–23)		Poor (≤17)		M ± SD		*t*/*H* value		*P*-value	
*Gender*															
Male		33 (35.11)			42 (44.68)		17 (18.09)		2 (2.13)		27.21 ± 4.34		*t* = −1.398		.163
Female		52 (38.52)			60 (44.44)		22 (16.30)		1 (0.74)		28.01 ± 4.15				
*Family background*															
Rural			11 (20.75)		28 (52.83)		12 (22.64)		2 (3.77)		25.89 ± 4.31		*t* = 3.61		.0004
Non-rural			74 (42.05)		74 (42.05)		27 (15.34)		1 (0.57)		28.22 ± 4.08				
*Only-child status*															
Only child		41 (44.09)			38 (40.86)		12 (12.90)		2 (2.15)		28.37 ± 4.48		*t* = 2.03		.0431
With siblings		44 (32.35)			64 (47.06)		27 (19.85)		1 (0.74)		27.21 ± 4.01				
*High school academic performance*															
Top 10%		71 (81.61)			16 (18.39)		0 (0.00)		0 (0.00)		31.49 ± 2.26		*H* = 168.72		.0000
Top 10–20%		14 (22.22)			49 (77.78)		0 (0.00)		0 (0.00)		27.86 ± 1.93				
Top 20–30%		0 (0.00)			35 (60.34)		22 (37.93)		1 (1.72)		24.33 ± 2.41				
Below top 30%		0 (0.00)			2 (9.52)		17 (80.95)		2 (9.52)		20.62 ± 2.56				
*Parents’ education level*															
Bachelor’s degree or above		39 (43.82)			36 (40.45)		13 (14.61)		1 (1.12)		28.39 ± 4.37		*t* = 2.04		.0425
Below bachelor’s degree		46 (32.86)			66 (47.14)		26 (18.57)		2 (1.43)		27.23 ± 4.11				

### 3.6. Multivariate regression analysis of the influence of demographic characteristics and critical thinking subdimensions on ethical decision-making

**Figure 2.**
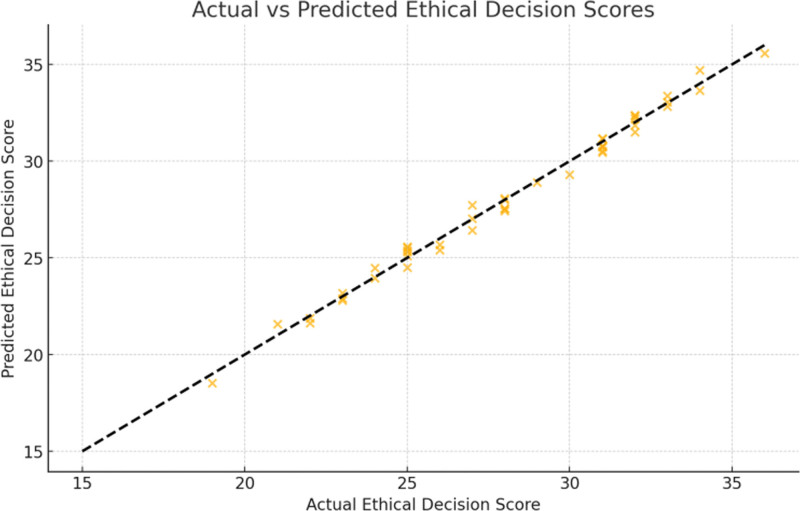
Predictive performance of the ridge regression model on ethical decision-making scores.

To further explore the impact of critical thinking on ethical decision-making when incorporating demographic characteristics, we employed a ridge regression analysis model. This model predicted ethical decision-making scores, including significant independent variables such as total CCTDI-CV score, family background, only-child status, high school academic performance, and parents’ education level. The model used regularization to reduce the impact of multicollinearity. Table 4 presents the variables that significantly influenced ethical decision-making scores, and the model demonstrated good predictive performance (Fig. 2).

**Table 4:** Significant variables influencing ethical decision-making scores (ridge regression analysis).

Variable	Coefficient	Standard error	*t*-Value	*P*-value
Intercept	16.178	0.316	51.342	<.001
Total CCTDI-CV score	0.0574	0.001	86.944	<.001
Family background (urban/rural)	0.3003	0.026	11.536	<.001
Only-child status	−0.006	0.054	−0.138	.891
High school academic performance	2.7704	0.025	111.145	<.001
Parents’ education level	−0.0124	0.054	−0.24	.811
*R* ^2^	0.9932			
*F*	6552.00			

CCTDI-CV = Chinese version of the California Critical Thinking Disposition Inventory.

## 4. Discussion

Cultivating medical students with high levels of critical thinking skills is particularly important in pediatric medical education. Strong critical thinking is not only crucial for clinical diagnosis and academic success but also lays the foundation for wise ethical decision-making in future clinical practice. Current research on the critical thinking abilities and ethical decision-making skills of students specializing in pediatrics is still limited, especially in the context of Chinese medical education.

This study, conducted with 229 pediatric students at Chongqing Medical University, found a significant correlation between critical thinking dispositions and ethical decision-making abilities. Most students exhibited positive critical thinking dispositions and also scored highly in ethical decision-making. Specifically, analysis ability, systematicity, and cognitive maturity were highly correlated with the total ethical decision-making scores, showing significant positive impacts. The results confirm the importance of critical thinking skills in medical ethical decision-making, particularly in the complex and sensitive context of pediatric medicine.

The total CCTDI-CV score played a decisive role in ethical decision-making abilities, with family background and high school academic performance also exerting some influence on these abilities. The findings underscore the necessity of integrating robust critical thinking training into pediatric medical education to enhance ethical decision-making skills, thereby improving the overall quality of healthcare services provided by future pediatricians.

This survey found that pediatric students generally exhibited good critical thinking dispositions, although 37.55% of the students were in an ambivalent state, indicating significant room for improvement in critical thinking skills. In terms of ethical decision-making abilities, over 80% of the students were rated as excellent or good, reflecting a high level of ethical judgment and decision-making skills. This could be attributed to the strengthened focus on medical ethics education.^[15]^ However, a small proportion of students showed fair or poor performance, suggesting the need for further targeted training and guidance to ensure that all students possess strong ethical decision-making abilities.

This study found that higher levels of ethical decision-making were associated with higher scores in critical thinking dispositions. Critical thinking skills help students systematically analyze and judge complex ethical situations, leading to more reasonable decisions.^[7]^ This result emphasizes the importance of fostering critical thinking skills in medical education, as they are not only crucial tools for academic research but also foundational for making wise ethical decisions in clinical practice. The positive correlations between the scores of each CCTDI-CV subdimension, total scores, and ethical decision-making scores further confirm the significant role of critical thinking in ethical decision-making.

Analytic ability, systematicity, and cognitive maturity had significant positive impacts on ethical decision-making, with cognitive maturity showing the highest correlation. This indicates that students need to possess systematic thinking and analytical skills, as well as a mature cognitive level, to make ethical decisions in complex clinical situations.^[15]^ Cognitive maturity involves not only the accumulation of knowledge but also the development of emotional and moral aspects,^[16]^ which are central to ethical decision-making. Systematicity helps students conduct orderly and comprehensive analyses in complex situations, which is particularly important for handling multiple and potentially conflicting ethical issues.^[17]^

This study also found that, in addition to the decisive influence of critical thinking abilities on ethical decision-making skills, students from different family backgrounds exhibited differences in ethical decision-making. This may be related to family educational environments, resources, and support. Educational policies can focus on students from various family backgrounds, providing targeted support and resources to help them achieve better performance in ethical decision-making. Students from rural areas scored lower than those from non-rural areas, possibly due to relatively insufficient educational resources in rural areas and less training in critical thinking and ethics education.^[18]^

Moreover, students with better high school academic performance performed better in ethical decision-making, reflecting the influence of academic background on students’ thinking abilities and ethical judgment,^[19]^ as well as their access to more educational resources and attention before entering university.^[20]^ These factors may also indirectly influence ethical decision-making abilities by affecting the development of students’ critical thinking and ethical values. This suggests the need to pay attention to students’ growth environments and academic performance to comprehensively enhance their ethical decision-making abilities.

This study has several limitations. Firstly, although the study included undergraduate students majoring in pediatrics, the relatively small number of students in this specialty and the focus on a single institution limit the generalizability of the conclusions. Future research should recruit larger and more representative samples of clinical medical students, including those from different specialties and institutions, to conduct comparative studies across various grades, specialties, and institutions. This approach would enhance the generalizability of the findings. Secondly, this study employed a cross-sectional design, which provides a snapshot of the current state but does not capture changes over time. Future research should consider longitudinal and intervention studies to develop effective methods for enhancing critical thinking skills and ethical decision-making abilities, and to explore the effectiveness of different teaching methods in improving these skills. Such studies would provide a deeper understanding of how these abilities evolve and how educational interventions can be optimized.

## 5. Conclusion

This study revealed the current status of critical thinking and ethical decision-making abilities among pediatric students at Chongqing Medical University and identified critical thinking abilities as a key factor influencing ethical decision-making abilities. These findings provide valuable references for medical educators, emphasizing the importance of critical thinking training in ethical education. Future medical education should focus more on cultivating multidimensional critical thinking skills to enhance students’ ethical decision-making abilities, thereby improving the overall quality of healthcare services. The results of this study can broaden the perspectives of pediatric medical educators globally, providing valuable international insights and references.

## Acknowledgments

We appreciate the cooperation of all participants during the study period. We appreciate Professor Liang, the biostatistician, for his assistance with the statistical analysis.

## Author contributions

**Data curation:** Hongxing Dang, Shaojun Li.

**Formal analysis:** Hongxing Dang.

**Funding acquisition:** Hongxing Dang.

**Investigation:** Hongxing Dang, Shaojun Li.

**Methodology:** Jing Li.

**Project administration:** Jing Li.

**Resources:** Jing Li.

**Supervision:** Jing Li.

**Validation:** Hongxing Dang, Shaojun Li.

**Visualization:** Hongxing Dang.

**Writing – original draft:** Hongxing Dang.

**Writing – review & editing:** Jing Li.
